# Delayed rupture of common carotid artery following rugby tackle injury: a case report

**DOI:** 10.1186/1749-7922-3-14

**Published:** 2008-03-21

**Authors:** Nainoor Thakore, Saleh Abbas, Peter Vanniasingham

**Affiliations:** 1 Department of Surgery, Middlemore Hospital, Auckland, New Zealand; 2 Department of Vascular Surgery, Middlemore Hospital, Auckland, New Zealand

## Abstract

**Background:**

Common Carotid Artery (CCA) is an uncommon site of injury following a blunt trauma, its presentation with spontaneous delayed rupture is even more uncommon and a rugby tackle leading to CCA injury is a rare event. What makes this case unique and very rare is combination of all of the above.

**Case presentation:**

Mr H. presented to the Emergency Department with an expanding neck haematoma and shortness of breath. He was promptly intubated and had contrast CT angiography of neck vessels which localized the bleeding spot on posteromedial aspect of his Right CCA. He underwent emergency surgery with repair of the defect and made an uneventful recovery post operatively.

**Conclusion:**

Delayed post traumatic rupture of the CCA is an uncommon yet potentially life threatening condition which can be caused by unusual blunt injury mechanism. A high index of suspicion and low threshold for investigating carotid injuries in the setting of blunt trauma is likely to be beneficial.

## Case report

Our patient is a 31 year old male teacher, who was brought in by ambulance to the Emergency Department. Two weeks earlier he had sustained an injury to the right side of his neck due to a rugby tackle, with a fierce "hand off" to the jaw extending his neck and rotating it to the left. He continued to play the game without any ill effects. A week prior to admission he was seen by his family physician and given an antibiotic for "swollen gland" in the right side of his neck. On the day of presentation he had sudden onset of right sided neck swelling with pain in the neck and shortness of breath.

His vitals were stable (Pulse 54, Respiratory Rate 24 and O_2 _saturation of 100%). He was conscious with GCS of 15/15. There was an obvious right sided neck swelling with trachea deviated to the left without any stridor. There was no neurological deficit or any evidence of Horner's syndrome. Our initial clinical impression was that he had a ruptured aneurysm.

**Figure 1:**
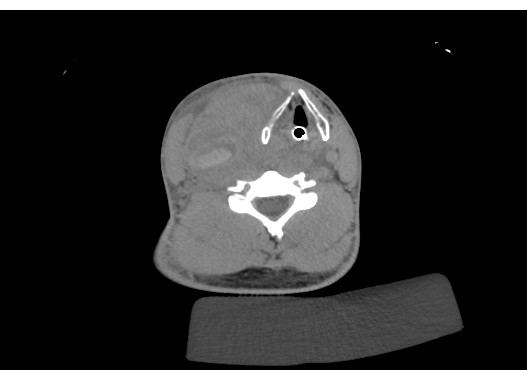
CT angiogram, transverse section showing the haematoma with marked displacement of airway to the left (endotracheal tube in situ).

**Figure 2:**
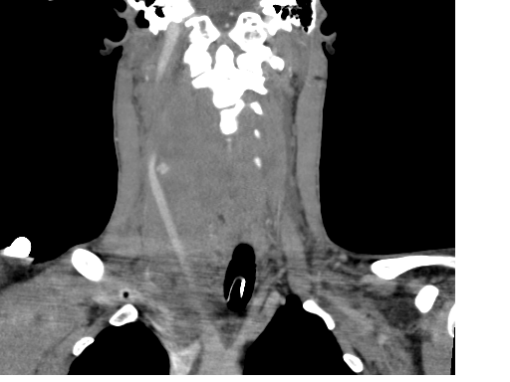
**CT angiogram, coronal section showing active leak of contrast from the right common carotid artery**. Note the marked displacement of airway to the left.

He was promptly intubated in resuscitation rooms and taken for CT scanning. CT angiogram of the neck vessels showed active extravasation of contrast from his right CCA (Fig. 1) resulting in the formation of a large haematoma extending superiorly into the parapharyngeal space and inferiorly into the superior mediastinum. It resulted in compression and marked displacement of the airway to the left (Fig. 2). The thyroid gland was also displaced and Internal Jugular Vein was effaced due to compression. Our impression was that he had ruptured pseudo aneurysm of the Right CCA and he was brought forward for emergency surgery.

**Figure 3:**
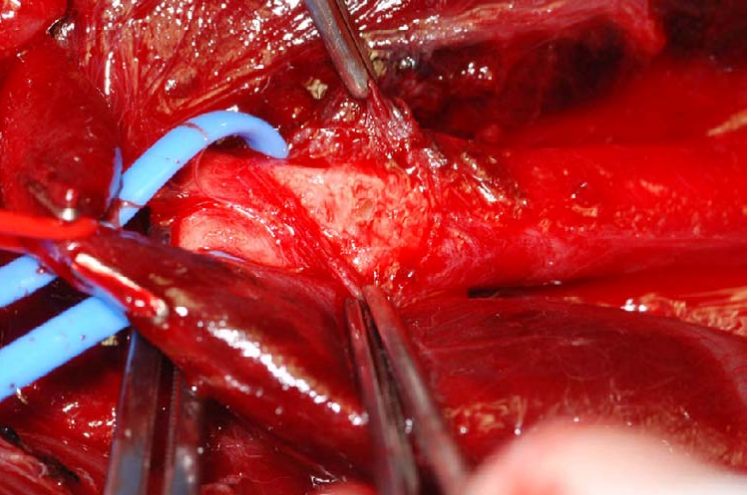
Intra operative picture showing (from above below): forceps holding the adventitia, Right Common Carotid Artery with localized perforation and Internal Jugular Vein.

His neck was opened on the right side using a lazy S incision. On opening his platysma, there was a bulging haematoma which was rapidly expanding. Control of the Right common carotid was obtained proximally at the level of sternal head of sternomastoid by deflecting it, and distally at the level of bifurcation of common carotid. Omohyoid muscle was also divided for access. He was heparinised and the right CCA was clamped above and below the site of injury. Haematoma site was exposed by reflecting the internal jugular vein laterally and taking its multiple branches medially between clips. Vagus nerve was identified and protected. The point of perforation was in the posteromedial aspect of the artery, with surrounding adventitia appearing somewhat ragged, suggesting a stretch injury (Fig. 3). This appearance was consistent with rupture of a pseudo aneurysm. The arterial perforation site was extended in a cranio-caudal fashion to expose normal vessel wall. Examination of rest of the arterial wall appeared relatively normal with no evidence of true or mycotic aneurysm. Primary closure of the extended defect was carried out with polypropylene 6-0 suture in two layers (first layer – full thickness wall, second – periadventitia as reinforcement). The sternal head of sternocleidomastoid muscle was reattached.

## Discussion

Blunt injury to the carotids represents about 3 to 10% of all carotid artery injuries with more than 90% involving the Internal Carotid Artery, often distally. It comprises of 0.08 to 0.33% of all traumatic blunt injuries. It is associated with mortality rates ranging from 5 to 43% and amongst the survivors only 20 to 63% of patients have good neurologic outcomes [1]. Causes of blunt injury include Motor vehicle accident, sports related injuries, strangulation, chiropractic manipulation and assault amongst others. Sports associated with carotid artery injury include Ice Hockey, Skiing, Horse riding, Rugby and Golf. Its occurrence due to a rugby tackle is rare. The mechanism of injury in blunt carotid trauma could be direct blow, hyperextension of neck with contralateral rotation of head, blunt intraoral trauma and skull base fracture [2].

In a patient with blunt trauma, carotid injury is easily missed as the clinical presentation is overshadowed by significant intra cranial injuries, effects of intoxication and other injuries if present. As many as half of the patients with carotid arterial injury show no signs of cervical trauma or neurological deficit at presentation [1]. In 43% of patients the diagnosis is missed until a neurological deficit manifests, with an average delay of up to 53 hours from injury to definitive diagnosis [3].

Clinical presentation is influenced by the extent of arterial damage. Intimal damage can cause dissection and subsequent thrombosis, which may lead to complete occlusion of artery. This usually presents as TIA's or stroke. Often there is a lucid interval between the injury and appearance of neurologic symptoms. This is usually of less than 24 hours [4]. Damage to tunica media with intact adventitia leads to pseudoaneurysm formation. Presentation can be delayed for several months to years, and is usually with a pulsatile mass with or without pressure symptoms, TIA's or stroke from embolism. Patients with partial or complete transection bleed into the neck. Severe damage of the artery is catastrophic and is usually fatal whereas minor bleeds can present with haematoma and pressure symptoms. Some of the blunt carotid injury patients present with Carotid – Cavernous fistula. Partial or complete Horner's syndrome may result from damage to cervical sympathetic fibres around the carotid artery.

More and more cases of carotid trauma are being discovered due to greater use of imaging tools, however due to low overall incidence of significant blunt carotid injury, routine screening is not considered practical [1]. Catheter Angiography is the gold standard diagnostic procedure in evaluating vascular trauma. It can be combined with intervention if suitable expertise is available. MR angiography gives additional information on brain damage but it requires the patient to be stable and compliant. It is time consuming, not readily available in all centres and the degree of definition may not be adequate to detail subtle injury. CT angiography is 98.6% sensitive and 100% specific, but can underestimate subtle lesions such as intimal flaps [5]. It is being increasingly used in acute trauma as it can be done rapidly and gives useful additional information on the state of adjacent structures and viscera. Once again the patient needs to be stable. USS while lacking sensitivity and being operator dependent can still be a useful screening tool. It has the advantage of being cheap and easily repeatable – ideal for long term follow up. Unfortunately it is not particularly good at imaging distal ICA which is the most commonly affected site in blunt trauma.

The extent of artery damage has been Graded from I – V [1]. Grade I = mild injury, Grade II = dissection or haematoma with luminal stenosis, Grade III = Pseudoaneurysm, Grade IV = occlusion and Grade V = transection. Grading helps in guiding management and determining prognosis. Management of blunt carotid artery injury depends on the type and extent of the injury. Patients with intimal tear, stable dissections and TIA's can be managed conservatively, with anticoagulation. Scans of the carotid artery repeated at regular intervals help in detecting progression and guiding early intervention. Established stroke may only need observation. Patients with symptomatic pseudoaneurysms, those with large symptomatic hematoma and/or ongoing bleeding would need surgical repair or endovascular stenting [6].

Endovascular covered stents are finding an increasing use in trauma patients in centres where the required expertise and infrastructure is available as they have obvious advantage in terms of morbidity. Some of the indications include deployment for emergency control of haemorrhage, enlarging dissections despite anticoagulation therapy, pseudoaneurysms, if the patient is unfit/has contraindications for surgery and for surgically inaccessible sites (e.g. high zone III injuries). Risks associated with its use include rupture of the vessel, stenosis or occlusion, thrombosis and distal embolization amongst others. Surgical repair remains the Gold Standard in managing rupture of the carotid artery. It can take the form of simple suturing for small defects and patch repair or bypass grafting for larger ones. Emergency surgery in the setting of acute haemorrhage can be very challenging; however it can be a life saver.

Our patient is an extremely unusual case highlighting injury to an unlikely site sustained by a rare mechanism. This case illustrates that carotid injuries can happen in unusual settings. It is possible that he had a "herald bleed" when he saw his family physician with a "gland in the neck" one week after the trauma and this injury could have been picked up at that stage by imaging. As a high proportion of carotid injury patients present without neurological deficit many cases are missed earlier on. A low threshold of imaging the carotids for injury in the setting of suspected trauma is prudent, especially as it can lead to serious sequelea including neurological deficit and death.

## Authors' contributions

NT collected data, obtained patient consent, drafted, revised and finalized the manuscript. SA edited the manuscript. PV conceived of the case report, supervised the drafting and editing of manuscript and provided valuable intellectual guidance. All authors read and approved the final manuscript.

## Consent

Patient has provided informed consent to publish case report, CT images and clinical photographs in the WJES.

## Funding

No financial contribution from any individual or organisation. Case report compiled by voluntary effort of the authors.
